# Electrophysiological Changes in Resting-State EEG Following REAC BWO-G_B Neurobiological Modulation in Healthy Adults: A Spectral and Multivariate Exploratory Study

**DOI:** 10.3390/brainsci16060549

**Published:** 2026-05-22

**Authors:** Sergio Brasil, Alessandra Renck, Sigride Thome-Souza, Jean Faber, Arianna Rinaldi, Vania Fontani, Wellingson Silva Paiva, Salvatore Rinaldi

**Affiliations:** 1 Hospital das Clínicas da Faculdade de Medicina da Universidade de São Paulo—USP, São Paulo 05403-000, Brazil; wellingsonpaiva@yahoo.com.br; 2 International Scientific Society of Neuro Psycho Physical Optimization with REAC Technology (SONC) Brazilian Branch, São Paulo 04029-000, Brazil; alerenck@alumni.usp.br; 3 Video Electroencephalography Monitoring Unit, Department and Institute of Psychiatry, University of São Paulo Medical School, São Paulo 04029-000, Brazil; maria.sigride@hc.fm.usp.br (S.T.-S.); jeanfaber@gmail.com (J.F.); 4 Department of Reparative and Regenerative Medicine, Rinaldi Fontani Institute, 50144 Florence, Italy; ari@irf.it (A.R.); vfontani@irf.it (V.F.); 5 Department of Adaptive Neuro Psycho Physio Pathology and Neuro Psycho Physical Optimization, Rinaldi Fontani Institute, 50144 Florence, Italy; 6 Research Department, Rinaldi Fontani Foundation, 50144 Florence, Italy

**Keywords:** radioelectric asymmetric conveyer, REAC, neurobiological modulation, EEG spectral analysis, multivariate EEG analysis, cortical oscillatory dynamics, cortical connectivity

## Abstract

**Highlights:**

**What are the main findings?**

- A standardized REAC BWO-G_B protocol was associated with reproducible changes in resting-state EEG patterns in healthy adults.
- Post-intervention EEG showed increased low-frequency spectral power, descriptive multivariate separability between baseline and post-intervention conditions, and a spatially differentiated redistribution of temporal synchronization.

**What are the implications of the main findings?**

- The observed findings support the presence of a reproducible electrophysiological pattern associated with the intervention under the conditions examined.
- These results provide a rationale for larger sham-controlled studies designed to assess specificity, mechanism, and potential functional relevance.

**Abstract:**

Background: Radio Electric Asymmetric Conveyer (REAC) neurobiological modulation is proposed as an approach designed to interact with endogenous bioelectrical processes involved in cortical regulation. However, its electrophysiological correlates in physiologically preserved neural systems remain insufficiently characterized. The present study explored whether a standardized REAC Brain Wave Optimization Gamma (BWO-G_B) protocol is associated with measurable changes in resting-state EEG activity in healthy adults. Methods: Nine neurologically healthy participants completed a standardized REAC BWO-G_B protocol consisting of 18 sessions administered over six consecutive days. Resting-state EEG recordings were obtained before and after the intervention. Spectral power was analyzed across the 1–100 Hz range. Multivariate organization of cortical activity was explored using Principal Component Analysis (PCA) and Canonical Discriminant Analysis (CDA), with CDA used only as a descriptive visualization of within-dataset multivariate organization. Cross-correlation analysis was applied to evaluate changes in inter-regional temporal synchronization. Individual-level non-parametric testing (Wilcoxon signed-rank test) was conducted only to characterize within-subject directional spectral modulation across the recorded montage. Results: Post-intervention EEG recordings showed a consistent redistribution of spectral power across cortical regions, predominantly within frequencies below approximately 20 Hz. This pattern was observed across subjects at the individual level. Multivariate analysis revealed a dissociation between PCA, which showed partial overlap between conditions, and CDA, which descriptively showed within-dataset separability between baseline and post-intervention cortical states. Cross-correlation analysis indicated a spatially differentiated redistribution of temporal synchronization across cortical regions. At the individual level, descriptive Wilcoxon analyses indicated broadband spectral differences in seven of nine participants (*p* < 0.05), with consistent directional trends across all subjects; these *p*-values should not be interpreted as confirmatory statistical evidence. Conclusions: The findings indicate the presence of a reproducible electrophysiological pattern observed after completion of the REAC BWO-G_B protocol in healthy adults. The observed combination of spectral redistribution, descriptive multivariate organization, and changes in temporal synchronization is consistent with a structured post-intervention modification of cortical activity organization within the present dataset. However, given the exploratory design, small sample size, absence of a control condition, and absence of objective vigilance monitoring, these results should be interpreted cautiously and should not be considered as evidence of intervention-specific effects. Further controlled studies are required to determine specificity, underlying mechanisms, and potential functional relevance.

## 1. Introduction

Radio Electric Asymmetric Conveyer (REAC) neurobiological modulation has been proposed as an approach aimed at interacting with endogenous bioelectrical processes involved in the regulation of cortical activity. Unlike conventional neuromodulation techniques that directly induce neuronal excitation or inhibition, REAC-based protocols are conceptually positioned as supporting the intrinsic regulatory dynamics underlying oscillatory organization and large-scale neural coordination [1,2,3].

Resting-state electroencephalography (EEG) provides a well-established framework for investigating large-scale cortical dynamics, offering direct access to oscillatory activity across multiple frequency bands. Patterns of spectral distribution, temporal synchronization, and multivariate organization of EEG signals have been associated with functional states of cortical regulation and network integration [4,5,6]. In this context, quantitative EEG approaches allow the characterization of both amplitude-related changes and higher-order features of cortical activity organization [7].

Resting-state EEG has been widely employed as an outcome measure across multiple non-invasive neuromodulation modalities, including repetitive transcranial magnetic stimulation and transcranial electrical stimulation, where it has documented intervention-associated modifications in oscillatory power, cortical excitability, and large-scale network organization in healthy adults [8,9,10]. This established methodological tradition provides a framework within which EEG-based assessment of REAC neurobiological modulation can be contextualized.

Previous investigations involving REAC protocols have primarily focused on clinical populations characterized by dysregulated neural networks; in such contexts, both neuroimaging and electrophysiological studies have documented intervention-associated modifications in brain functional organization [1]. An in vivo electrophysiological investigation conducted at the National Research Council of Italy (CNR) documented measurable modulation of thalamocortical neural activity under controlled experimental conditions [3]. Rodent model studies conducted by independent research groups have further reported REAC-associated modulation of neuroinflammatory processes and behavioral outcomes in models of neurodegeneration.

However, the extent to which similar electrophysiological patterns may be observed in physiologically preserved neural systems remains less clearly defined.

The study of healthy individuals provides a controlled context for examining cortical dynamics in the absence of overt pathological alterations. This approach allows exploration of whether measurable electrophysiological changes may occur under standardized intervention conditions, without the confounding influence of disease-related network disruptions. At the same time, resting-state brain activity in healthy subjects is known to exhibit intrinsic variability and dynamic organization, reflecting ongoing adaptive regulatory processes.

Within the REAC protocol family, Brain Wave Optimization (BWO) protocols are designed to operate at the level of cortical oscillatory organization. Their proposed rationale is not limited to overt pathological conditions but extends to the modulation of electrophysiological patterns that may not manifest as clinical symptoms yet remain measurable at the level of neural dynamics. In this sense, the investigation of healthy subjects represents a relevant model for exploring the electrophysiological correlates associated with REAC BWO-G_B neurobiological modulation.

To situate the present investigation within the broader landscape of EEG-based neuromodulation research, Table 1 provides a structured comparison of selected studies employing quantitative EEG or neuroimaging approaches to assess the effects of non-invasive neurobiological modulation, including prior REAC investigations.

Exploratory designs with limited sample sizes are common in early-phase mechanistic EEG research across multiple neuromodulation modalities. The present study is aligned with this methodological tradition while acknowledging its specific limitations.

On this basis, the present study investigated resting-state EEG activity in neurologically healthy adults undergoing a complete standardized REAC BWO-G_B neurobiological modulation cycle. The analytical framework combined spectral analysis across an extended frequency range (1–100 Hz) with multivariate approaches, including Principal Component Analysis (PCA) [11] and Canonical Discriminant Analysis (CDA) [12], as well as cross-correlation analysis of temporal synchronization patterns.

The objective of this exploratory investigation was to determine whether completion of the standardized protocol was associated with reproducible post-intervention changes in electrophysiological patterns of cortical activity, and to examine whether such changes were more consistent with simple spectral modulation or with structured alterations in the multivariate organization of cortical dynamics. No assumptions were made regarding specific underlying mechanisms, and the study was not designed to establish causal, intervention-specific, or clinical effects, but rather to characterize signal-level patterns observed after the intervention under standardized experimental conditions.

## 2. Materials and Methods

### 2.1. Participants

Nine neurologically healthy volunteers (45–85 years; mean age 62.8 ± 16.5 years; median 58.5 years; 60% female) were enrolled, with no history of neurological, psychiatric, or major systemic disease, no prior head trauma with loss of consciousness, and no current use of psychoactive or neuroactive medications. All participants underwent medical screening to confirm eligibility and were instructed to avoid caffeine, alcohol, and sleep deprivation prior to EEG recordings. Enrollment was conducted on a voluntary basis, and no participant received financial compensation. All procedures were conducted in accordance with the Declaration of Helsinki and complied with international ethical standards for human research.

The sample size is consistent with exploratory EEG investigations in healthy participants, where the objective is to characterize electrophysiological patterns rather than estimate population effect sizes. The relatively broad and older age range of the sample should be considered when interpreting the spectral findings, since age-related differences may influence resting-state EEG characteristics [13,14,15].

### 2.2. Study Design

This investigation was designed as an exploratory electrophysiological study aimed at characterizing resting-state cortical signal changes associated with the intervention under physiological conditions rather than evaluating clinical efficacy.

Consistent with this objective, the study was not intended to assess therapeutic outcomes or symptom modification, but to document electrophysiological changes under carefully standardized intervention conditions.

Each participant underwent two EEG recordings: one at baseline and one immediately after completion of the neurobiological modulation cycle.

All nine participants completed the full protocol without protocol deviations or adverse events.

### 2.3. Rationale for Healthy Subjects

The choice of healthy individuals was methodologically deliberate. Investigating neurobiological modulation in non-pathological neural systems allows characterization of intrinsic cortical regulatory behavior without confounding effects of pathological network alterations, providing a methodologically clean context for exploratory electrophysiological assessment.

### 2.4. Neurobiological Modulation Intervention

All participants received a complete REAC Brain Wave Optimization Gamma (BWO-G_B) neurobiological modulation cycle using the REAC BENE 110 medical device (ASMED, Scandicci, Italy). The treatment consisted of 18 sessions administered over six consecutive days, as three daily applications of 15 min each, with a minimum interval of one hour between sessions. As with all REAC protocols, treatment parameters were factory pre-set and not modifiable by the operator, ensuring procedural standardization, reproducibility, and operator independence under standardized medical-device conditions. The Asymmetric Conveyer Probe was positioned according to BWO-G_B protocol specifications. The present study was not designed to establish a direct biophysical mechanism at the scalp or cortical tissue level, and no mechanistic inference is made from the intervention description.

### 2.5. EEG Recording and Preprocessing

EEG recordings were obtained in a controlled laboratory environment with participants in an awake, relaxed resting state, eyes closed, with instructions to minimize movement, facial muscle activity, and jaw tension. No objective vigilance monitoring, pupillometry, behavioral alertness task, or subjective sleepiness scale was included; this limitation was considered in the interpretation of low-frequency activity.

Scalp electrodes were positioned according to the international 10–20 system [16] using an 18-channel bipolar montage organized into three chains: left hemisphere (FP1–F3, F3–C3, C3–P3, P3–O1, FP1–F7, F7–T7, T7–P7, P7–O1), midline (Fz–Cz, Cz–Pz), and right hemisphere (FP2–F4, F4–C4, C4–P4, P4–O2, FP2–F8, F8–T8, T8–P8, P8–O2), ensuring full cortical coverage across major regions.

EEG signals were acquired using Nihon Kohden (Tokyo, Japan) EEG 1200 J/K digital equipment, with Neurofax QP-112AK version 11-50 and Neuroworkbench version 08-04, together with simultaneous video recording using a Sony^®^ Ipela camera (Sony Corporation, Tokyo, Japan). Data were sampled at 1000 Hz, allowing reliable spectral characterization up to 100 Hz. Acquisition settings included a sensitivity of 7 µV/mm, a time constant of 0.3 s, a high-frequency filter of 70 Hz, and a paper speed of 10 mm/s.

Artifact removal was performed by visual inspection of the EEG recordings, supported by simultaneous video monitoring, in order to exclude segments affected by movement, eye movements, muscle activity, or non-physiological environmental interference. Only artifact-free segments were included in the subsequent analyses.

Because EEG signals were recorded using an 18-channel bipolar montage and subsequently summarized at the level of cortical macro-regions, the spectral profiles shown in the regional grand-average plots should not be interpreted as equivalent to conventional referential occipital derivations. In particular, the visual prominence of the canonical posterior alpha peak may be attenuated by bipolar derivation geometry, regional averaging, and inter-individual spectral variability, even when posterior alpha activity is present at the individual channel level. The spectral displays are therefore intended to describe condition-related regional patterns within this specific analytical framework rather than to reproduce the morphology of standard referential occipital EEG traces.

Power Spectral Density (PSD) estimation was performed using Welch’s averaged modified periodogram method [17], with segmentation into 2 s windows, Hamming windowing to reduce spectral leakage, and overlap between consecutive segments to preserve temporal continuity. This approach ensured a balance between spectral resolution and estimator stability.

Spectral analysis was extended across the 1–100 Hz frequency range, enabling characterization of canonical EEG frequency bands as well as the transition between beta and gamma domains. Particular attention was devoted to minimizing and identifying potential sources of high-frequency contamination, including muscle artifacts and environmental electromagnetic interference. Power-line-related fluctuations (50–60 Hz) were explicitly identified and not interpreted as neurophysiological signals.

All preprocessing and analysis procedures were conducted using standardized signal-processing methods.

### 2.6. Quantitative EEG Analysis

Quantitative EEG analysis evaluated cortical activity across five anatomical macro-regions: central, frontal, parietal, temporal, and occipital cortices.

Spectral power was evaluated across the following canonical frequency bands: delta (1–4 Hz), theta (4–8 Hz), alpha (8–13 Hz), beta (13–30 Hz), and gamma (30–100 Hz) [15,16]. Particular analytical attention was directed to low-frequency components (delta and theta, below approximately 8 Hz, and, more broadly, the sub-20 Hz range), where the most consistent post-intervention modulation emerged, as well as to the spectral transition between beta and gamma domains (approximately 25–40 Hz).

PSD was computed for each region in both baseline and post-intervention conditions, at both the individual and group-averaged levels.

### 2.7. Multivariate Cortical State Analysis

To characterize cortical organization beyond regional spectral features, a multivariate analytical framework was implemented to explore potential differences in cortical state structure between baseline and post-intervention recordings. PCA [11] was used to examine variance-driven organization of the data, whereas CDA [12] was applied only as a descriptive visualization of condition-related organization within the observed dataset. The objective of this complementary approach was not to establish classification performance, inferential discrimination, or generalizability, but to compare different representations of multivariate signal structure. Cortical behavior was additionally visualized through three-dimensional state projections, allowing descriptive evaluation of spatial dispersion and separability across conditions. Future studies will integrate quantitative clustering indices and cross-validation procedures to further assess the robustness of these observations.

### 2.8. Statistical Considerations

Given the exploratory nature of this investigation, the analytical approach was designed to characterize reproducible patterns of electrophysiological modulation rather than to estimate population-level effect sizes or to perform confirmatory hypothesis testing. Accordingly, the analysis emphasized convergence across multiple complementary methods.

Spectral differences between pre- and post-intervention conditions were evaluated at the individual level using Wilcoxon signed-rank tests applied across EEG channels (*n* = 18 paired observations per subject). Because EEG channels are spatially correlated measurements from the same brain, they should not be considered fully independent biological observations. Accordingly, these tests were used only to characterize within-subject directional spectral consistency across the recorded montage. The resulting *p*-values should be interpreted descriptively and not as formal population-level statistical inference. Directional consistency of spectral changes was evaluated across subjects and cortical regions as convergent descriptive evidence. It is explicitly acknowledged that sign concordance across subjects does not constitute formal statistical inference and should not be interpreted as a substitute for group-level hypothesis testing.

Multivariate analyses (PCA and CDA) were used to explore the organization of cortical state space. CDA was applied as a descriptive tool to assess condition-related organization within the observed dataset, not as a predictive classifier or inferential model. It is important to emphasize that with the present sample size (*n* = 9) and the dimensionality of the EEG spectral data, CDA is inherently susceptible to overfitting. The observed separability should therefore not be interpreted as evidence of classification performance, intervention specificity, or generalizable discrimination beyond this dataset. No cross-validation was performed, which constitutes a recognized limitation of the multivariate analyses. Quantitative clustering metrics and cross-validation procedures are planned for future studies. Accordingly, CDA results are presented only as a descriptive visualization of structured separability within the observed dataset.

No correction for multiple comparisons was applied, as the analysis was not designed for confirmatory inference. This choice implies that the reported *p*-values do not control the family-wise error rate and that false-positive findings cannot be excluded. This limitation is explicitly acknowledged. All statistical results should be interpreted within the context of an exploratory framework and should not be considered as confirmatory evidence of effect.

Formal confirmatory statistical analysis, including larger sample sizes, cross-validation, Bonferroni or FDR correction for multiple comparisons, quantitative clustering metrics (e.g., silhouette index), and pre-registered hypotheses, is planned for future sham-controlled studies.

### 2.9. Ethics

The study was conducted in accordance with the Declaration of Helsinki and approved by the Ethics Committee of Hospital das Clínicas da Faculdade de Medicina da Universidade de São Paulo (USP) under CAAE 84791824.8.0000.0068 and registered in clinicaltrials.gov under the number NCT07373366. All participants provided written informed consent prior to participation.

### 2.10. Data Availability

Data supporting the findings of this study are available under institutional data governance policies. Further access may be granted upon reasonable request to the corresponding author, subject to ethical compliance and data-sharing regulations.

## 3. Results

### 3.1. Spectral Analysis

**Figure 1:**
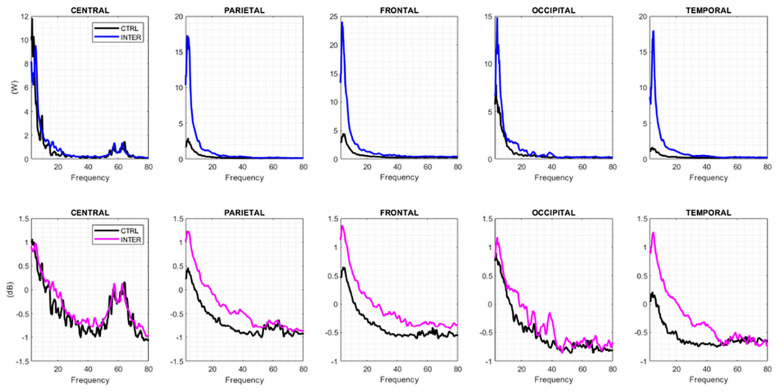
Grand Average Power Spectral Density (PSD) by Cortical Region. Curves represent averaged PSD computed across all participants for the pre-intervention baseline condition (Control/CTRL, black line) and the post-intervention condition (Intervention/REAC/INTER, blue–magenta line). Both conditions were recorded from the same participants in a within-subject design; “Control” therefore denotes the pre-intervention recording. The upper row displays spectral power on a linear scale, whereas the lower row presents the same data on a logarithmic scale (dB), facilitating visualization of low-amplitude differences. The five columns correspond to the Regions of Interest: Central, Parietal, Frontal, Occipital, and Temporal. Notably, the post-intervention condition (INTER) shows a consistent increase in spectral power predominantly within frequencies below approximately 20 Hz. Because the spectra are derived from a bipolar montage and displayed as macro-regional grand averages, they should not be interpreted as equivalent to conventional referential occipital recordings, and the visual prominence of the posterior alpha peak may therefore be attenuated in this representation.

Spectral analysis showed a consistent redistribution of power across all cortical regions, predominantly within frequencies below approximately 20 Hz (Figure 1).

At the group-averaged level, the post-intervention condition exhibited higher spectral power values than baseline, particularly within the delta and theta ranges. This pattern was observed across the central, parietal, frontal, occipital, and temporal regions. A further difference between conditions was visible near the beta–gamma transition range [18], where the spectral profiles showed a reproducible inflection pattern rather than a uniform broadband increase.

At the individual-subject level, the same general directional trend was observed in all participants, although with expected inter-individual variability in absolute spectral amplitude and profile morphology. These regional grand-average spectra should be interpreted within the analytical framework adopted in this study, namely, bipolar derivation and macro-regional averaging. Accordingly, the morphology of the occipital spectral profile is not directly equivalent to that expected from conventional referential posterior derivations, and the apparent prominence of posterior alpha activity may be reduced in the regional plots despite its presence at the individual or channel level.

### 3.2. Three-Dimensional Cortical State Projections

**Figure 2:**
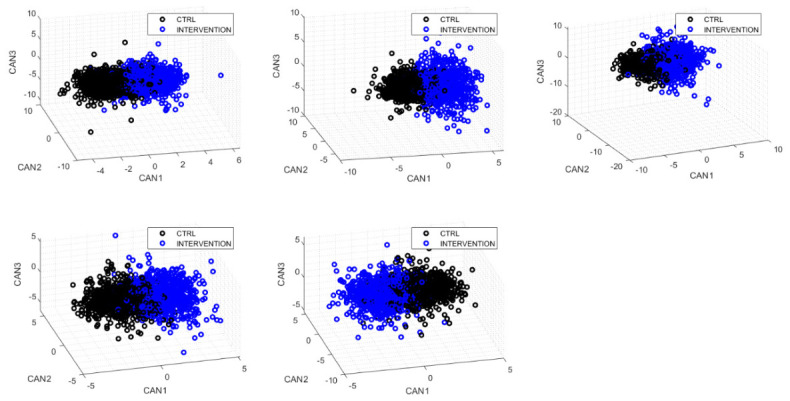
Canonical Discriminant Analysis (CDA) in Three-Dimensional Space. Three-dimensional projection of scores from the first three canonical functions (CAN1, CAN2, CAN3) derived from multivariate analysis of variance (MANOVA) applied to spectral data. Each point represents an EEG sample, color-coded by condition: baseline/Control (CTRL, black circles) and post-intervention (INTER, blue circles). The five panels correspond to the cortical regions analyzed. Visual inspection shows separability between baseline and post-intervention observations within the analyzed dataset, with the most evident separation in the frontal and parietal regions.

Three-dimensional cortical state projections showed visual differentiation between baseline and post-intervention conditions across all cortical regions. In several regions, post-intervention point distributions appeared less dispersed and more spatially compact than baseline distributions, particularly in the frontal and parietal areas (Figure 2).

This pattern was further explored using CDA, which provided a descriptive visualization of the separation between baseline and post-intervention observations within the analyzed dataset across all five cortical regions. The degree of visual separation was most evident in the frontal and parietal regions. These observations are not interpreted as classification performance or generalizable discrimination.

**Figure 3:**
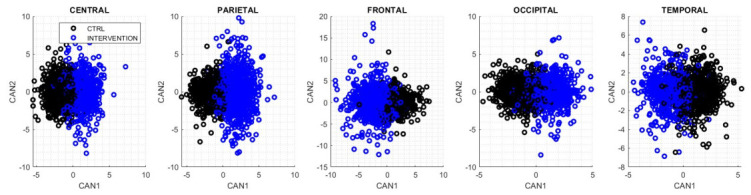
Canonical Discriminant Analysis (CDA)—Two-Dimensional Cluster Projection. Two-dimensional scatter plot of scores along the first two canonical discriminant functions (CAN1, CAN2) derived from multivariate analysis of variance (MANOVA) applied to EEG spectral data. Each point represents an EEG sample, color-coded by condition: baseline/Control (CTRL, black circles) and post-intervention (INTER, blue circles). The five panels correspond to the five cortical regions of interest (central, parietal, frontal, occipital, temporal). Across all regions, the two conditions show visual separability in canonical discriminant space. Given the exploratory nature of the study, the limited sample size, and the absence of cross-validation, these findings are presented only as a descriptive visualization of separability within the observed dataset and not as evidence of classification performance or generalizability.

To further illustrate the same multivariate structure, a two-dimensional projection of the first two canonical discriminant functions (CAN1 vs. CAN2) is presented in Figure 3.

These observations are presented as descriptive evidence of an organized multivariate configuration within the observed dataset, rather than as evidence of classification performance or generalizable discrimination. Although nominal statistical values were obtained for the canonical functions, these were not interpreted inferentially given the exploratory design, limited sample size, and absence of cross-validation.

### 3.3. PCA/CDA Dissociation and Individual Spectral Profiles

In contrast to CDA, Principal Component Analysis (PCA) showed only partial differentiation between baseline and post-intervention conditions, with overlap persisting across cortical regions.

This difference between PCA and CDA indicates that the two analytical approaches captured distinct aspects of the data structure. Whereas PCA primarily reflected a variance-driven organization, CDA provided a descriptive representation of condition-related separability within the present dataset. In this sense, the observed dissociation suggests that the differences between conditions were not fully captured by the main variance components alone, although this interpretation remains descriptive and dataset-bound.

**Figure 4:**
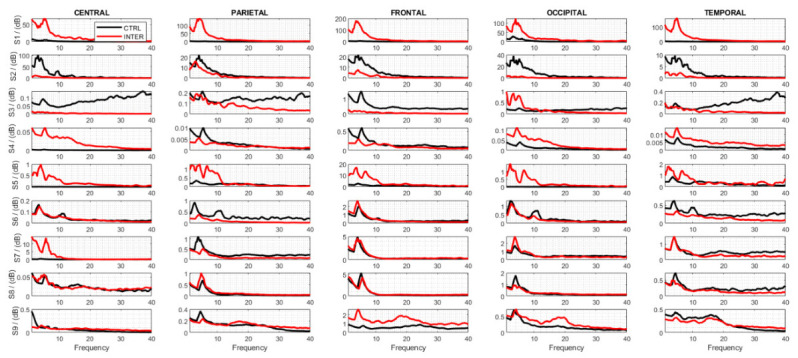
Individual Subject Power Spectral Density by Region. Matrix plot displaying individual subject PSD for nine subjects (rows S1–S9) distributed across five cortical regions (columns). Black curves represent the Control group; red curves represent the Intervention (INTER) group. Y-axes are in absolute scale (dB) with independent scaling per subject to accommodate inter-individual amplitude variability. The frequency axis is displayed up to 40 Hz to optimize visualization of the low-frequency components where the principal spectral modulation is concentrated; the complete 1–100 Hz spectral analysis is presented in Figure 1. The consistency of the post-intervention pattern (INTER > CTRL) is observed across subjects and is consistent with the group-averaged spectral findings.

Spectral analysis extended up to 100 Hz provided additional detail on the frequency distribution of the observed modulation (Figure 4).

Across subjects and cortical regions, post-intervention spectra generally showed higher power values than baseline in the lower frequency range, particularly below approximately 20 Hz. This pattern was consistently observed despite variability in absolute amplitudes between individuals.

A localized increase near 50–60 Hz was also observed and was attributed to environmental power-line interference. This component was not considered in the neurophysiological interpretation.

### 3.4. Cross-Correlation Analysis

**Figure 5:**
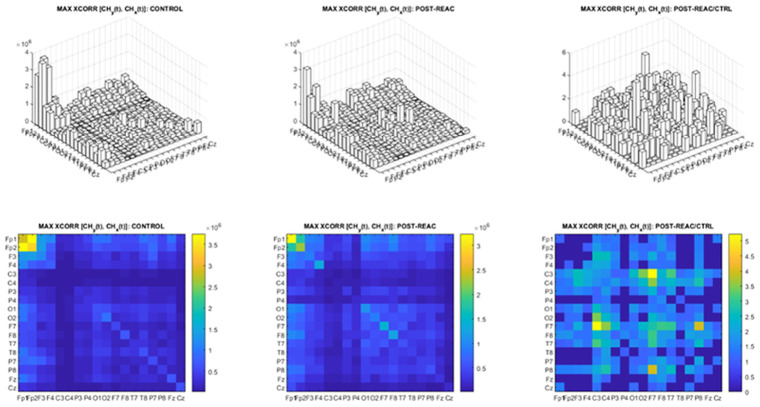
Maximum Cross-Correlation Analysis (Max XCorr). Upper panel: Three-dimensional bar plots representing the median maximum cross-correlation value across all 18 channels for the Control group (**left**), Post-REAC group (**center**), and the between-group ratio (**right**). Lower panel: Corresponding heatmaps. The color scale indicates correlation intensity (higher values in yellow). The ratio map (**right**) highlights specific connections where the intervention increased temporal correlation (values > 1), particularly outside the polar frontal region.

Cross-correlation analysis of temporal synchronization across all 18 EEG channels is presented in Figure 5.

The group-level results, visualized through three-dimensional bar plots and heatmaps, showed that changes in maximum cross-correlation values after the intervention were not uniformly distributed across channel pairs. The ratio map indicated relatively greater increases in temporal synchronization in connections involving central, parietal, and occipital channels, whereas frontal polar regions showed comparatively smaller changes.

This spatially differentiated pattern indicates that the observed changes in temporal synchronization were regionally heterogeneous rather than globally uniform across the scalp.

### 3.5. Per-Subject Spectral Band Analysis and Non-Parametric Statistical Assessment

To characterize the spectral modulation at the individual level, Wilcoxon signed-rank tests were applied for each subject to compare baseline and post-intervention total spectral band power across the 18 EEG channels. Because these channels are spatially correlated and cannot be considered independent biological observations, this analysis was used only as a descriptive assessment of within-subject directional consistency.

**Figure 6:**
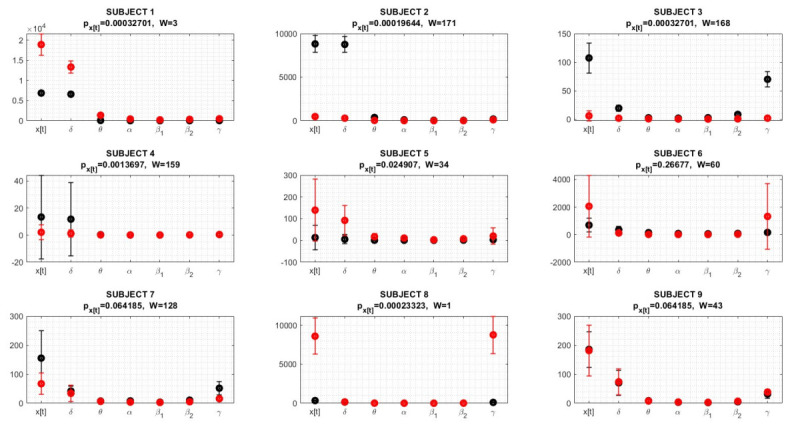
Per-Subject Total Spectral Band Power Comparison and Wilcoxon Signed-Rank Test Results. Each panel corresponds to one of the nine subjects (S1–S9). The x-axis displays the broadband signal x[t] and the canonical EEG frequency bands (delta, theta, alpha, beta-1, beta-2, gamma). The y-axis represents median total spectral power per band. Black circles indicate the baseline/Control condition (CTRL), and red circles indicate the post-intervention condition (INTER); error bars represent variability across the 18 EEG channels. The Wilcoxon signed-rank test was applied across the 18 channels as paired observations (*n* = 18 per subject), comparing total band power between conditions; the W statistic ranges from 0 to 171 for *n* = 18. Each panel title reports the W statistic and nominal *p*-value for the broadband comparison (p_x[t]_). These values are reported descriptively because EEG channels are spatially correlated and were not modeled as independent biological observations. Across subjects, the delta band showed the largest absolute post-intervention increase in spectral power.

Results are presented in Figure 6, which shows for each subject the median total spectral power for the broadband signal and for the canonical EEG bands, together with the corresponding Wilcoxon W statistic and nominal *p*-value for the broadband comparison. These values are reported descriptively and should not be interpreted as confirmatory statistical evidence, but rather as an indication of within-subject directional consistency.

Seven of the nine subjects showed nominal broadband differences at *p* < 0.05. Although the remaining subjects did not meet this nominal threshold, their spectral profiles followed the overall group trend.

Across subjects, the delta band showed the largest absolute increase in post-intervention spectral power, followed by theta, in agreement with the group-averaged spectral findings.

### 3.6. Overall Pattern of Findings

Taken together, the analyses identified a reproducible post-intervention electrophysiological pattern. This pattern was characterized by redistribution of spectral power predominantly at lower frequencies, descriptive multivariate separability within the observed dataset, and a regionally differentiated modification of temporal synchronization patterns.

## 4. Discussion

The present exploratory study identified a reproducible pattern of resting-state EEG changes following completion of a standardized REAC BWO-G_B neurobiological modulation cycle in healthy adults. Across complementary analytical approaches, the post-intervention condition was associated with redistribution of spectral power predominantly within frequencies below approximately 20 Hz, descriptive multivariate separability between baseline and post-intervention cortical states, and a spatially differentiated redistribution of temporal synchronization. Taken together, these findings support the presence of a measurable post-intervention electrophysiological pattern under the standardized conditions examined.

The first relevant aspect of the present results is the consistency of the low-frequency spectral changes across subjects and cortical regions. The post-intervention condition showed higher spectral power values predominantly in the delta and theta ranges, with similar directional trends observed at both the group and individual levels. This finding indicates that the observed modulation was not restricted to isolated channels or a small subset of participants but instead emerged as a recurrent feature of the dataset. At the same time, the interpretation of this pattern requires caution.

In resting-state EEG, increases in slow-frequency activity may reflect different physiological conditions, including changes in vigilance, relaxation, reduced alertness, or adaptive shifts in cortical state. Therefore, the present data do not justify assigning a unique functional meaning to the observed low-frequency enhancement.

In particular, it must be explicitly acknowledged that the possibility of a non-specific reduction in arousal or an accumulation of fatigue across the 18-session protocol cannot be excluded. This interpretation is consistent with established neurophysiological literature associating delta and theta increases in healthy individuals with drowsiness, relaxed wakefulness, or reduced engagement [19,20], rather than with enhanced cortical coordination. Without continuous vigilance monitoring or a concurrent behavioral task, the present data cannot disambiguate between these alternatives. Age-related changes in resting-state EEG spectral characteristics, including shifts in alpha peak frequency and slow-frequency power redistribution, are well-documented in the neurophysiological literature and may contribute to the interpretation of baseline and post-intervention profiles in the present sample [13,14,15].

Several features of the dataset are, however, not fully consistent with a purely non-specific arousal account. First, the redistribution of temporal synchronization was spatially differentiated, with more prominent changes in central, parietal, and occipital regions and comparatively smaller modifications in frontal polar areas. A generalized drowsiness effect would be expected to produce a more diffuse topographic pattern. Second, the CDA, while acknowledged to be susceptible to overfitting, showed descriptive separability across all five cortical regions, suggesting an organized multivariate configuration rather than a purely random post-intervention pattern within this dataset. Third, convergent findings were reported using the same BWO-G protocol in a clinically distinct population, arguing against a purely session-specific or setting-dependent explanation. These observations collectively suggest that while a non-specific arousal reduction cannot be ruled out, the multivariate structure of the post-intervention EEG may reflect contributions beyond simple drowsiness. Verification in a sham-controlled design with continuous vigilance monitoring is necessary to resolve this interpretive ambiguity.

At the same time, the observed electrophysiological pattern is not entirely isolated [2]. A recent retrospective case series using the same REAC BWO-G protocol and multimodal neurophysiological assessment reported convergent post-intervention changes, including redistribution of spectral power, increased symmetry, and reorganization of cortical source activity across multiple subjects exposed to chronic stress [2]. Although those findings were obtained in a clinically dysregulated population and under similarly uncontrolled conditions, they provide contextual support for the reproducibility of structured post-intervention EEG configurations across different subject groups.

Nevertheless, the low-frequency spectral redistribution observed here did not occur in isolation. The associated multivariate and synchronization findings suggest that the observed phenomenon may not be fully described as a simple global amplitude increase. In particular, PCA showed only partial overlap reduction between baseline and post-intervention conditions, whereas CDA showed descriptive separability within the observed dataset across all cortical regions.

Within the limits of an exploratory design, this dissociation is of interest because it suggests that the post-intervention EEG pattern may involve a change in the organization of multivariate signal structure, rather than only a uniform rescaling of spectral power. This interpretation should remain descriptive and dataset-bound, especially given the limited sample size and the absence of cross-validation, but it supports considering the observed modulation as structured and worthy of further investigation.

The cross-correlation analysis adds a further level of information. Rather than indicating a homogeneous increase in inter-regional coupling, the results showed a spatially differentiated redistribution of temporal synchronization, with more evident changes involving central, parietal, and occipital regions and comparatively smaller modifications in frontal polar areas. This topographic heterogeneity argues against a purely uniform or nonspecific amplification effect and is more consistent with a structured reconfiguration of temporal relationships across channels. Even so, cross-correlation does not by itself establish functional improvement or a specific neurobiological mechanism, and the present observations should therefore be considered as signal-level correlates requiring further confirmation.

An additional element supporting the internal coherence of the results is the convergence between group-level observations and individual-level analyses. The per-subject Wilcoxon signed-rank tests showed nominal broadband differences in seven of nine participants, while the remaining subjects maintained the same general directional trend despite not reaching the nominal threshold. Because EEG channels are spatially correlated and were not modeled as independent biological units, these results should be interpreted as descriptive evidence of within-subject directional consistency rather than as formal inferential evidence. In resting-state EEG studies, such inter-individual variability is not unexpected, particularly in small exploratory samples [13]. Signal processing methodological choices, including spectral estimation approach and derivation type, are also known to influence inter-individual variability in resting-state EEG power estimates [21]. For this reason, the present results are better interpreted in terms of reproducible pattern consistency than in terms of uniform response magnitude.

The present study was intentionally conducted in healthy adults to examine electrophysiological correlates of the intervention in the absence of overt neurological or psychiatric pathology. This design offers a relatively controlled context for describing signal-level changes while reducing the confounding influence of disease-related cortical disorganization. At the same time, it imposes important interpretive limits. Because the participants were neurologically healthy and no behavioral or cognitive outcome measures were included, the present findings cannot be linked to clinical benefit, functional improvement, or symptom-related modulation. In addition, the relatively older age distribution of the sample may have influenced baseline spectral characteristics and should be considered when interpreting the findings [13,14,22]. The results, therefore, remain confined to the level of electrophysiological characterization.

The absence of a sham or control condition prevents causal attribution and does not allow exclusion of non-specific influences such as repeated measurement, adaptation to the recording environment, relaxation, expectancy, or changes in vigilance. This limitation is particularly relevant when interpreting increases in slow-frequency activity, since similar changes may also occur under non-interventional conditions. For this reason, the present findings should not be interpreted as definitive evidence of specificity, but rather as documenting a reproducible post-intervention electrophysiological pattern that warrants verification in appropriately controlled studies.

The small sample size further limits the strength and generalizability of the conclusions. In addition, although the multivariate analyses showed descriptive condition-related separability within the observed dataset, the limited sample size and lack of cross-validation preclude any inference regarding robustness beyond the present sample. In particular, the CDA findings should not be interpreted as evidence of classification performance or broad generalizability, but only as descriptive evidence of separability within this dataset.

An additional methodological point concerns the graphical representation of the spectral data. Because the EEG analysis was based on bipolar derivations and macro-regional averaging, the grand-average occipital spectra should not be interpreted as direct equivalents of standard referential posterior traces, and this may reduce the visual prominence of the canonical alpha peak in healthy subjects.

A further point concerns mechanistic interpretation. The current data document measurable changes in resting-state EEG patterns following the intervention, but do not establish the biological pathway through which such changes may have occurred. The present study was not designed to demonstrate a defined mechanism of action, and no such claim is made here.

Nevertheless, these findings can be contextualized within a broader body of mechanistic evidence on REAC technology. In vivo electrophysiological studies conducted at the CNR documented that REAC stimulation modulates thalamocortical neural activity in controlled animal preparations, including under experimentally perturbed conditions [3]. Independent rodent model studies reported REAC-associated changes in neuroinflammatory markers and behavioral outcomes in models of neurodegeneration. At the human level, a randomized double-blind fMRI investigation showed long-lasting modifications in brain activation patterns following a single REAC pulse [1]. While none of these studies directly addresses the BWO-G_B protocol in healthy adults, they collectively support the biological plausibility of intervention-associated modulation of cortical activity organization and provide a mechanistic framework within which the present EEG findings may be interpreted.

The specific pathway by which the BWO-G_B protocol may interact with cortical dynamics in healthy adults remains to be defined. Future mechanistic studies combining quantitative EEG with source localization, functional connectivity analysis, and multimodal neurophysiological assessment will be necessary to characterize the neural substrates of the observed signal-level patterns.

Within these limits, the study also has strengths. EEG acquisition and preprocessing were standardized, artifact management was explicitly applied, the intervention protocol was fixed and operator-independent, and the findings showed convergence across spectral, multivariate, synchronization, and individual-level analyses. This convergence does not establish specificity, but it supports the presence of a coherent and structured post-intervention EEG pattern that warrants further controlled investigation. These limitations are also broadly consistent with the current early-stage literature in this area, which remains largely exploratory and case-based [2].

Future research should therefore focus on sham-controlled and randomized designs, larger cohorts, pre-registered hypotheses, and quantitative validation procedures for multivariate analyses, including cross-validation and complementary clustering metrics. The integration of behavioral, cognitive, and multimodal neurophysiological outcomes will also be necessary to determine whether the observed EEG changes have significance beyond signal-level modulation.

In summary, completion of a standardized REAC BWO-G_B protocol in healthy adults was associated with a reproducible pattern of resting-state EEG changes characterized by low-frequency spectral redistribution, descriptive multivariate separability of cortical states, and spatially differentiated modification of temporal synchronization. These findings should be interpreted cautiously within the limits of an exploratory uncontrolled study, but they support the rationale for further controlled investigations into the electrophysiological correlates of this intervention.

## 5. Conclusions

In this exploratory study, completion of a standardized REAC BWO-G_B neurobiological modulation cycle was followed by a reproducible pattern of resting-state EEG changes in healthy adults. The observed findings included redistribution of spectral power predominantly within frequencies below approximately 20 Hz, descriptive multivariate separability between baseline and post-intervention cortical states, and a spatially differentiated redistribution of temporal synchronization across cortical regions.

These results suggest the presence of a structured post-intervention modification of electrophysiological signal organization within the observed dataset. The present study should therefore be regarded as a descriptive and hypothesis-generating contribution that identifies a signal-level phenomenon requiring confirmation under controlled conditions.

Further confirmatory studies with larger samples, sham-controlled designs, objective vigilance monitoring, cross-validation of multivariate analyses, and correction for multiple comparisons are required to determine whether the electrophysiological pattern observed here reflects a specific effect of the intervention and to clarify its potential biological and translational relevance.

## Figures and Tables

**Table 1:** Comparative overview of selected studies using quantitative EEG or neuroimaging to assess non-invasive neurobiological modulation effects.

Study	Modality	Population	*N*	Design	Primary Outcome	Control
Rinaldi et al., 2014 [1]	REAC (pulse)	Healthy adults	20	RCT double-blind	fMRI brain activation	Sham
Zippo et al., 2015 [3]	REAC (BWO)	Rat (in vivo)	—	Controlled lab (CNR)	Thalamocortical electrophysiology	Yes
Rinaldi et al., 2025 [2]	REAC BWO-G	Chronic stress (clinical)	Case series	Retrospective	Multimodal EEG + source imaging	No
Present study	REAC BWO-G_B	Healthy adults	9	Exploratory pre-post	Spectral + multivariate EEG	No

RCT = randomized controlled trial; — = animal study (sample size not applicable).

## Data Availability

The datasets generated and analyzed during the current study are maintained by the Hospital das Clínicas da Faculdade de Medicina da Universidade de São Paulo (USP) and may be made available upon reasonable request, subject to institutional approval.
